# Skate, overtravel, and contact force of tilted triangular cantilevers for microcantilever-based MEMS probe technologies

**DOI:** 10.1038/s41598-022-23973-5

**Published:** 2022-11-12

**Authors:** Steve Arscott

**Affiliations:** University of Lille, CNRS, Centrale Lille, University Polytechnique Hauts-de-France, UMR 8520-IEMN, 59000 Lille, France

**Keywords:** Engineering, Electrical and electronic engineering, Mechanical engineering

## Abstract

Microfabricated chip-edge microcantilevers are commonly used as surface probes, e.g. in near-field microscopy. Such probes normally function in the low-deflection regime, where their behaviour is very well understood and documented. In contrast, when microcantilevers are used for applications such as electrical testing probes, their deflection can be somewhat higher, taking them into the less well understood high-deflection regime of microelectromechanical systems (MEMS). Here, a scalable model for the relationship between the skate, overtravel, and resulting tip contact force in tilted triangular cantilevers—which are bending with high deflection and in contact with a flat surface—is presented. The model is tested experimentally using macroscopic triangular cantilevers—the experimental results agree well with the proposed model. The findings enable a practical solution for zero-skate in tapered MEMS probes to be suggested. It is hoped that the findings may be of use for probe engineers involved with on-wafer testing and designers of emerging MEMS micro cantilever-based probes.

## Introduction

**Figure 1:**
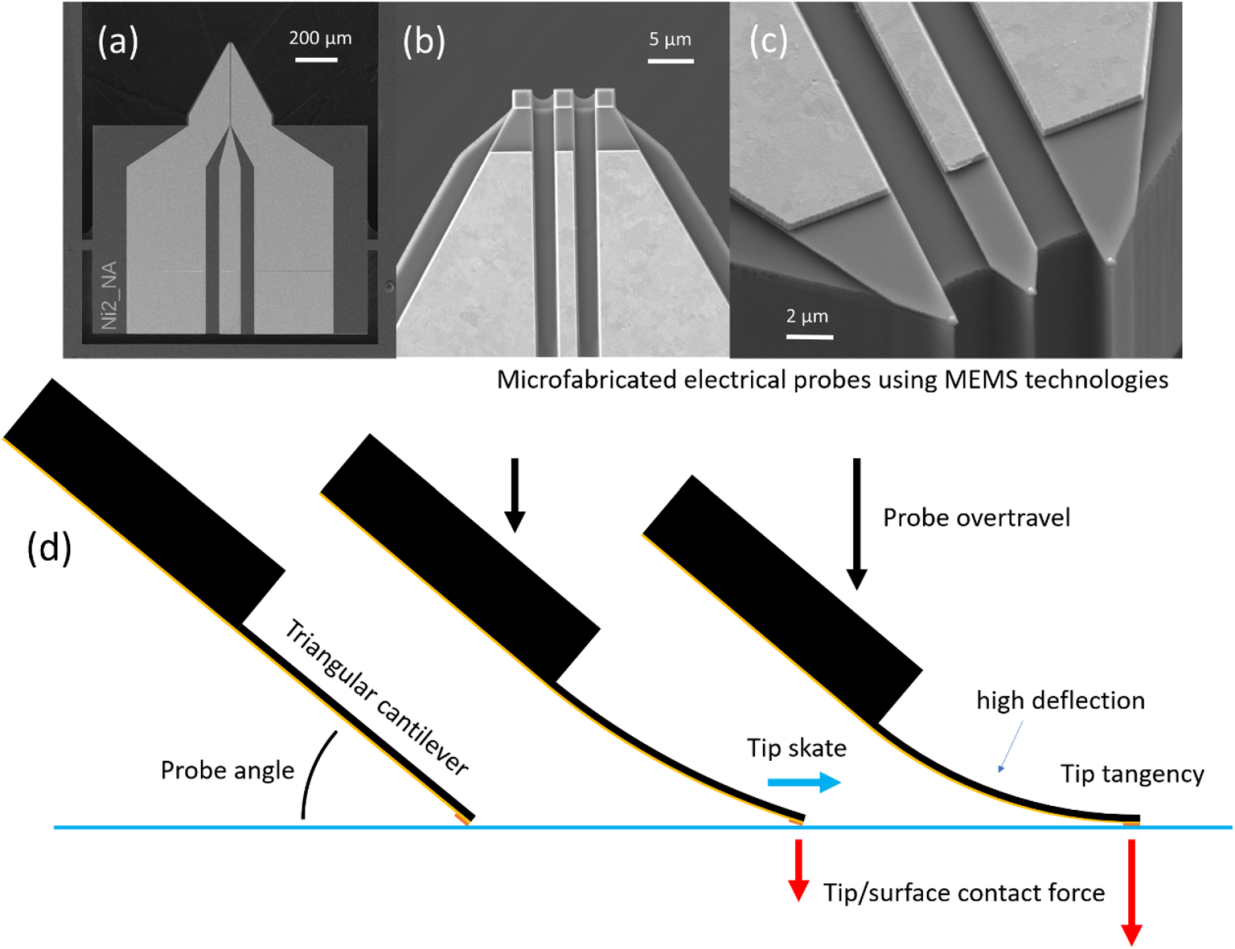
Microfabricated electrical probes using MEMS technologies which incorporate a microcantilever. SEM images showing (**a**) a silicon support chip and protruding ‘chip-edge’ triangular microcantilever, (**b**) and (**c**) zooms on tip showing the contact pads having micrometre (2 µm × 2 µm gold pads) and sub-micrometre size (200 nm × 200 nm gold pads). (**d**) A schematic illustration of how an imposed downward overtravel (black arrows) achieves a necessary contact force (red arrows) for a low resistivity contact but at the expense of a tip skate (blue arrow) across the surface du to bending of the microcantilever.

Tiny deformable microfabricated chip-edge microcantilevers are very useful for a whole range of surface probing techniques^1^. For example, they are a key element for near-field microscopy—where their mechanical behaviour in the low-deflection regime is very well understood and documented, indeed essential, for the interpretation of results^2–4^. The tip deflection of such cantilevers used in near-field microscopy is counted in nanometres—this is very small compared to their length, which is typically tens and hundreds of micrometres. In contrast, if microcantilevers are used as vehicles for other miniature probing techniques, such as emerging miniature electrical testing probes^5–8^, their deflection can be somewhat higher, e.g. due to the potential necessity of a larger contact force to achieve low electrical resistance contacting. Figure 1a–c gives examples of MEMS-based electrical probes fabricated using silicon microtechnology which can create long, micrometre-thick cantilevers. For such probes, their bending can take potentially them into the high deflection configuration—where their mechanical behaviour is somewhat less well understood and documented. In high deflection, the tip deflection could be as high as half the length of the cantilever. A tilted, microcantilever-based probe in contact with a surface deforms mechanically as a vertical movement known as ‘overtravel’ is imposed. The result of this bending is (i) a lateral movement of the tip across the contacting surface known as ‘skate’ and (ii) the generation of a contact force between the tip and the surface—see Fig. 1d. The relationship between the overtravel, the skate, and the contact force depends on the shape and stiffness of the cantilever, as well as its tilt angle relative to the underlying surface. This relationship has been explored for cantilevers in the low-deflection regime^9,10^ and described by the author for a rectangular cantilever undergoing high deflection^11^. In contrast, flat, tapered triangular shaped microcantilevers are favourable for on-wafer testing^8^ due to their ease of positioning and a tapering which allows wide, low electrical resistance tracks to be relayed to small tip contacts—this is apparent in Fig. 1a–c; e.g. the gold pad contacts in Fig. 1c are 200 nm by 200 nm. The relationship between the skate, the overtravel, and the contact force of a tilted cantilever/surface configuration depends on the bending of the cantilever which, in turn, depends on its specific shape. A flat tapered, triangular cantilever deflects differently from its rectangular counterpart—meaning that the overtravel/skate/contact force relationship will be different. This relationship, between skate, overtravel, and contact force for a flat triangular cantilever, is described and investigated here. The contact force is an important parameter as it, in part, governs the quality of a metal-to-metal electrical contact^12^ in the case of an electrical probe. The surface stress generated on the bending cantilever is also important as it will govern the maximum imposable overtravel of the probe—beyond which the probe is likely to fail. Tip/surface tangency may also be of some importance in some applications to enable any metallic contact pads located at the probe tip to be parallel to the underlying contacting surface—along with the contact force, this geometry may also have implications in the minimum achievable contact resistance of the probe. The idea of zero-skate, via overtravel compensation^11^, also applies to triangular cantilevers—the findings point to a practical solution for this. Being able to predict the above issues is important for the engineer of MEMS microcantilever-based probes functioning in high-deflection—this is the objective of this paper.

## Experimental results and discussions

### Fabrication of the triangular cantilevers

Dense, flat polystyrene sheets (Schulcz, Germany) were chosen to fabricate macroscopic triangular cantilevers for the study for three main reasons. First, the sheet thickness is very uniform—this is important as the mechanical properties of cantilevers are particularly sensitive to thickness. Second, it has a low density—meaning that bending due to gravity of macroscopic cantilevers will be negligible. Thirdly, centimetre-scale lateral dimensions and millimetre-scale thickness enable polystyrene triangular cantilevers to be fabricated having stiffnesses in the same range as equivalent silicon cantilevers having micrometre dimension—this is due to the ratio of the elastic moduli of polystyrene and silicon equal to approximately 0.01^13,14^. This last point enables—at least in principle due to scaling—the findings to be useful for triangular MEMS microcantilever probe applications.

**Figure 2:**
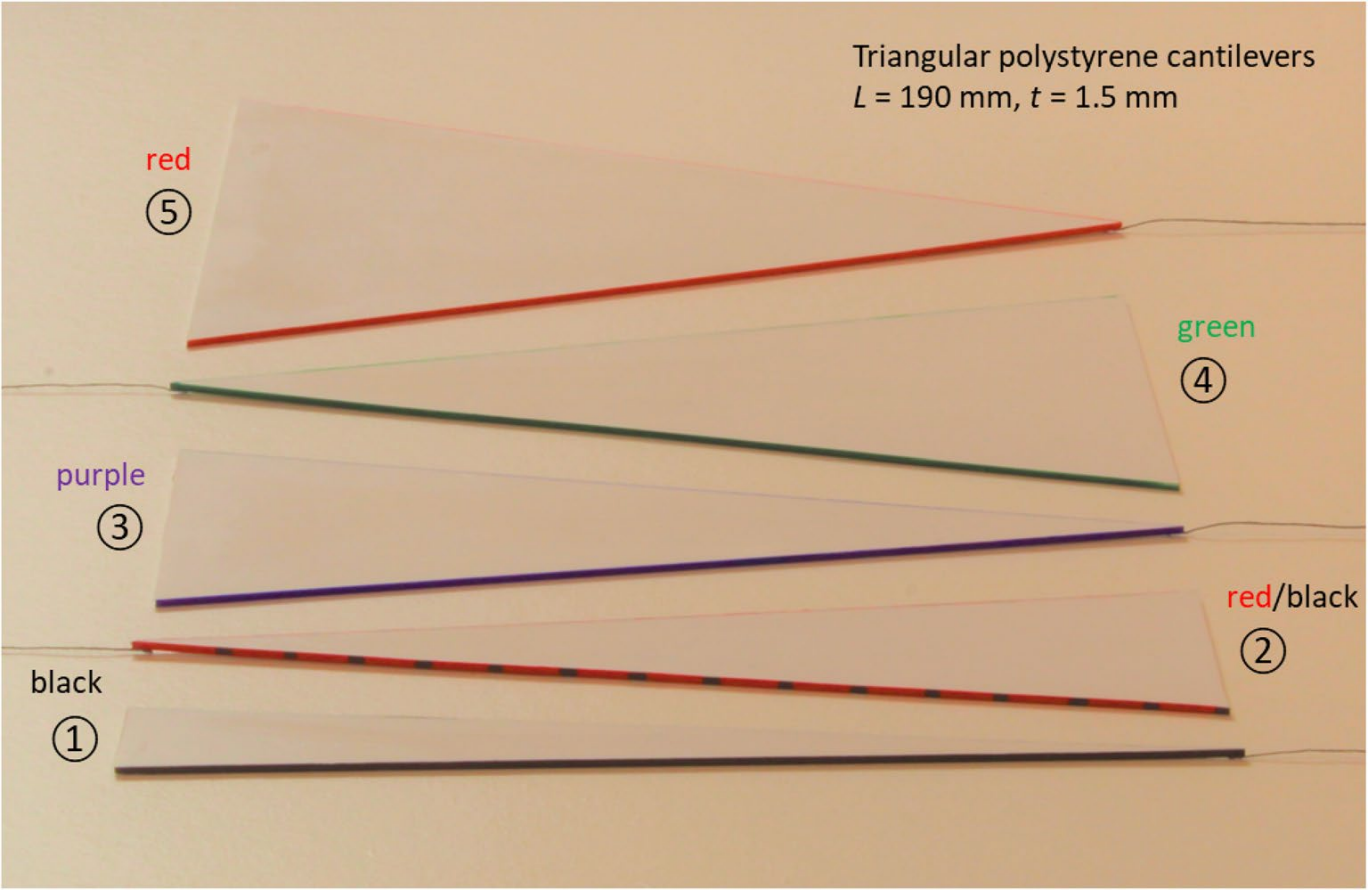
A photograph showing the five flat triangular macroscopic cantilevers fabricated for the study. The cantilever material is dense polystyrene. The cantilever thickness is 1.5 mm, the length is 190 mm, and the base widths (from bottom to top) are 22 mm (black), 47 mm (red/black), 69 mm (purple), 95 mm (green), and 141 mm (red).

The thickness of the polystyrene sheet used to fabricate the triangular cantilevers was determined to be 1516 ± 5 µm (measured using a precision thickness meter—Mitutoyo, Japan). The lateral cutting accuracy was estimated at ± 0.5 mm. Thus, the five polystyrene triangular cantilevers used for the study have a length $L$ equal to 190 ± 0.5 mm, a thickness $t$ of 1516 ± 5 µm, and a base width $b$ equal to 22 mm, 47 mm, 69 mm; 95 mm, and 141 mm—all with an error of ± 0.5. Figure 2 shows photographs of the cantilevers manufactured for the study. The density of the polystyrene was measured to be 1042 ± 2 kg m^−3^. We will see that the elastic modulus of polystyrene was experimentally-determined to be 1.75 GPa—close to published data for very dense material^13^. Table 1 shows the calculated stiffnesses and weights of the triangular cantilevers for the five different base widths $b$.

**Table 1:** Information concerning the triangular cantilevers used in the study.

Colour code	Base width $b$ (mm)	Stiffness (N m^−1^)	Weight (g)	*η* (m) × 10^–9^
Black	22	3.3	3.3	1.86
Red/black	47	7	7.1	3.98
Purple	69	10.2	10.4	5.84
Green	95	14.1	14.3	8.05
Red	141	20.9	21.2	11.94

Edge colour code, triangular base width *b*, stiffness, weight, and the constant $\eta =b{t}^{3}/6{L}^{3}$ used in the study to normalize the tip/surface contact force. The length $L$ of the cantilevers is 190 mm and their thickness $t$ is 1.5 mm.

The flat polystyrene sheets 21 cm × 30 cm were cut using a sharp craft knife and a steel ruler into 5 isosceles triangles having a height of 190 mm and base widths of 22 mm, 47 mm, 69 mm, 95 mm, and 141 mm. Each cantilever edge was colour coded (see Table 1) to facilitate visibility and analysis of photographic data after experimentation. Each cantilever was fabricated to have an anchoring area at its base—this area had a depth of approximately 10 mm for each triangular cantilever.

### Skate versus overtravel relationship of tilted triangular cantilevers

**Figure 3:**
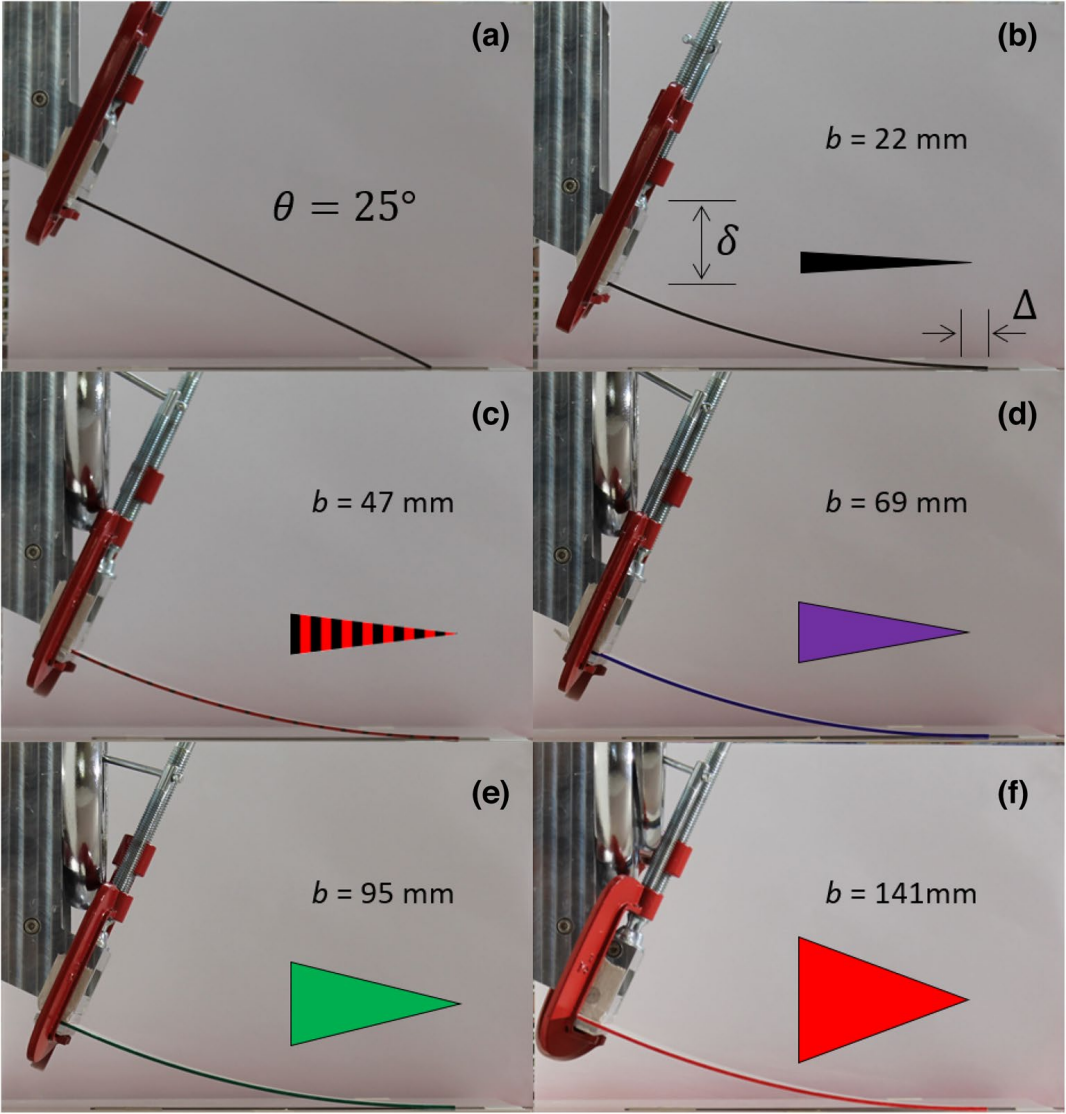
Photographs of the skate and overtravel of triangular cantilevers inclined at 25° to the horizontal. (**a**) A triangular cantilever in contact with the surface. (**b**–**f**) The triangular cantilevers are shown when the tip is tangent to the surface. In each case the length of the triangular cantilever is 190 mm.

**Figure 4:**
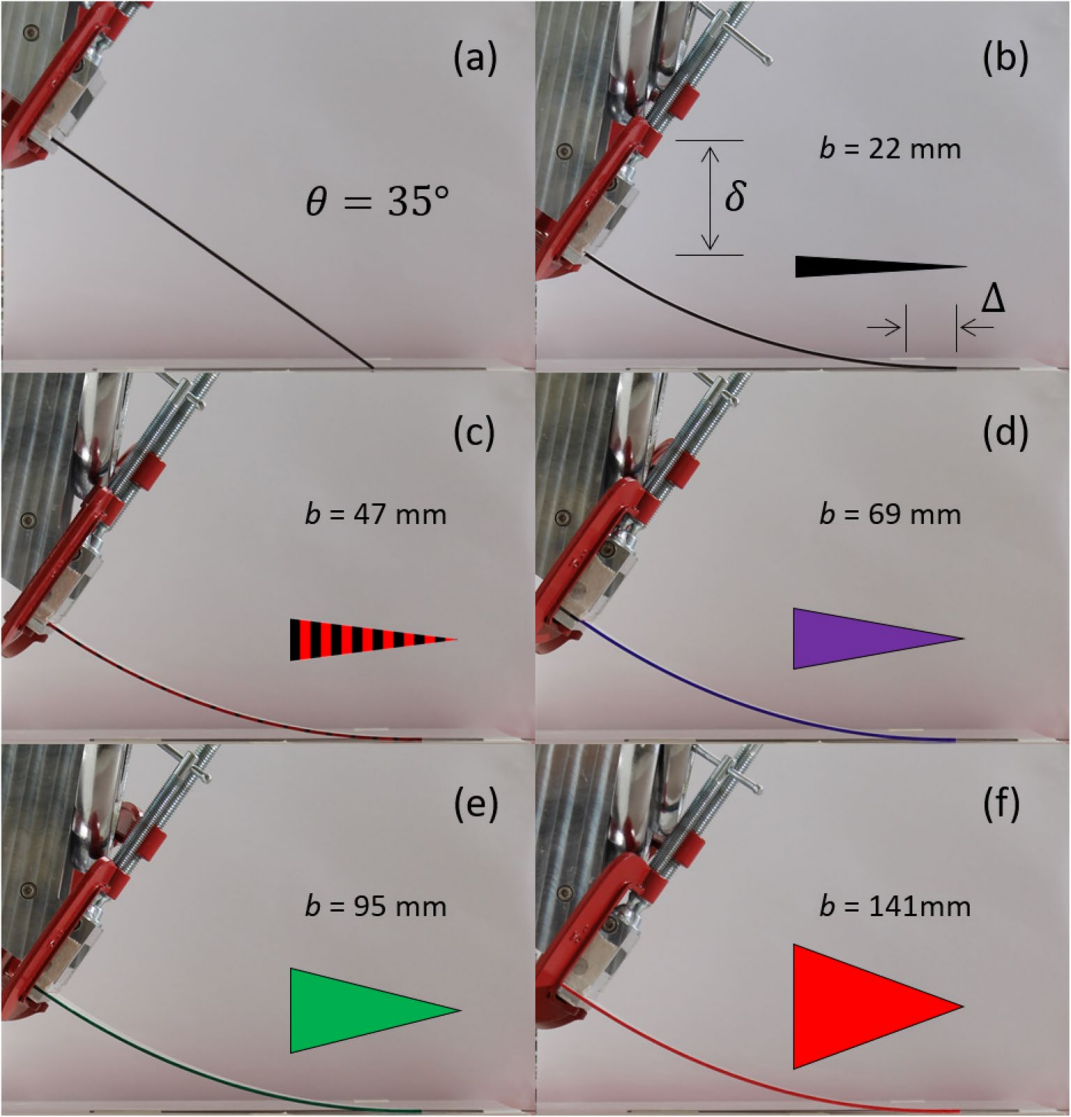
Photographs of the skate and overtravel of triangular cantilevers inclined at 35° to the horizontal. (**a**) A triangular cantilever in contact with the surface. (**b**–**f**) The triangular cantilevers are shown when the tip is tangent to the surface. In each case the length of the triangular cantilever is 190 mm.

Figures 3 and 4 show the experimental results of triangular cantilevers bending due to an imposed vertical overtravel. Two tilt angles are used; 25° (Fig. 3) and 35° (Fig. 4). The tip of the cantilever skates across the surface as the overtravel is increased. The results are shown for 5 cantilevers having different base widths varying from 22 to 141 mm. All skate versus overtravel experimental results in the form of photographs can be found in the Supplementary Information (see Movies 1–5).

**Figure 5:**
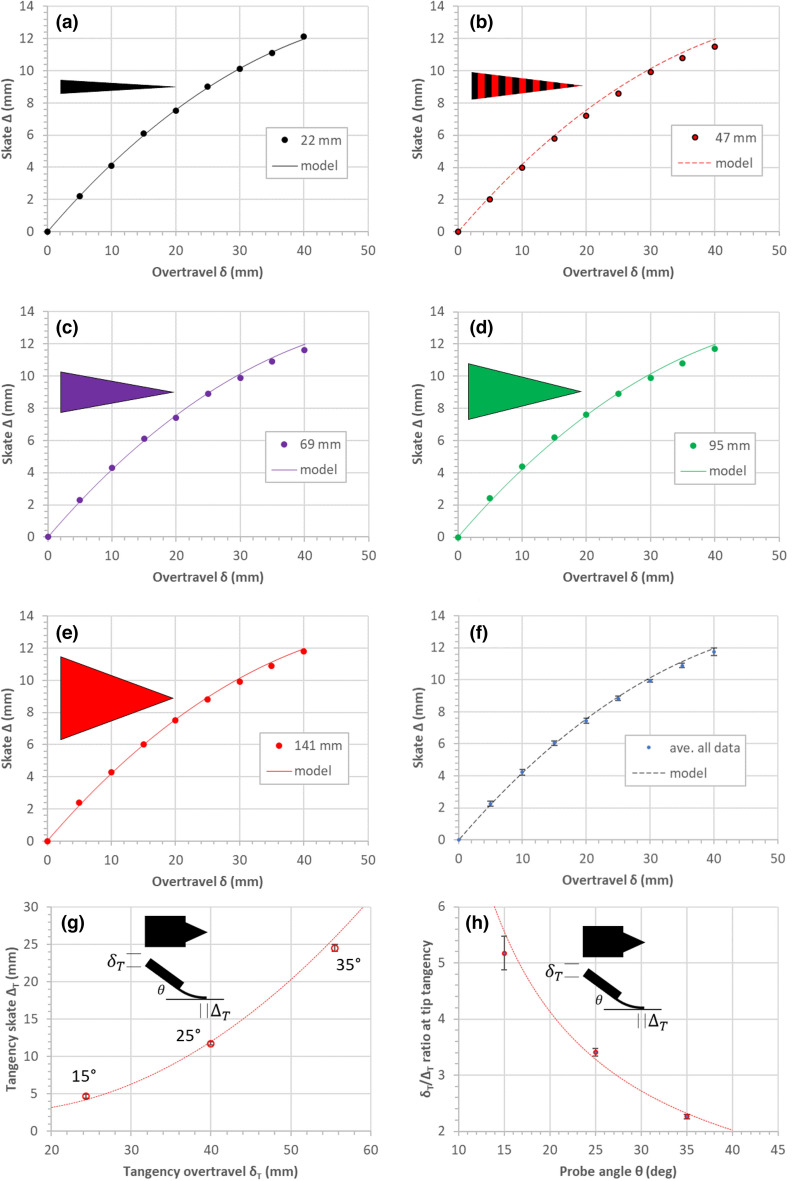
Experimental results of skate/overtravel of triangular cantilevers. (**a**–**e**) Skate versus overtravel for the five different triangulate cantilevers. (**f**) Averaged data plotted. (**g**) The tangency skate plotted as a function of tangency overtravel. (**h**) The tangency overtravel/tangency skate ratio plotted as a function of the cantilever tilt angle. The traces (solid and dashed) correspond to the mathematical model developed in “Methods”. The cantilever tilt (probe) angle is 25°.

Figure 5 shows plots of the data extracted from the overtravel/skate experiments. Note that the overtravel versus skate plots are very similar irrespective of the cantilever base width (a) to (e)—this is evident by the small error bars in Fig. 5f which plots all averaged data. Figure 5g plots the measured tangency skate versus the required tangency overtravel for three different cantilever tilt angles. Figure 5h plots the overtravel/skate ratio experimental data points versus the cantilever tilt angle. The dashed lines in Fig. 5g,h correspond to graphs generated by the modelling (see “Methods”).

### Contact force versus overtravel relationship of tilted triangular cantilevers

**Figure 6:**
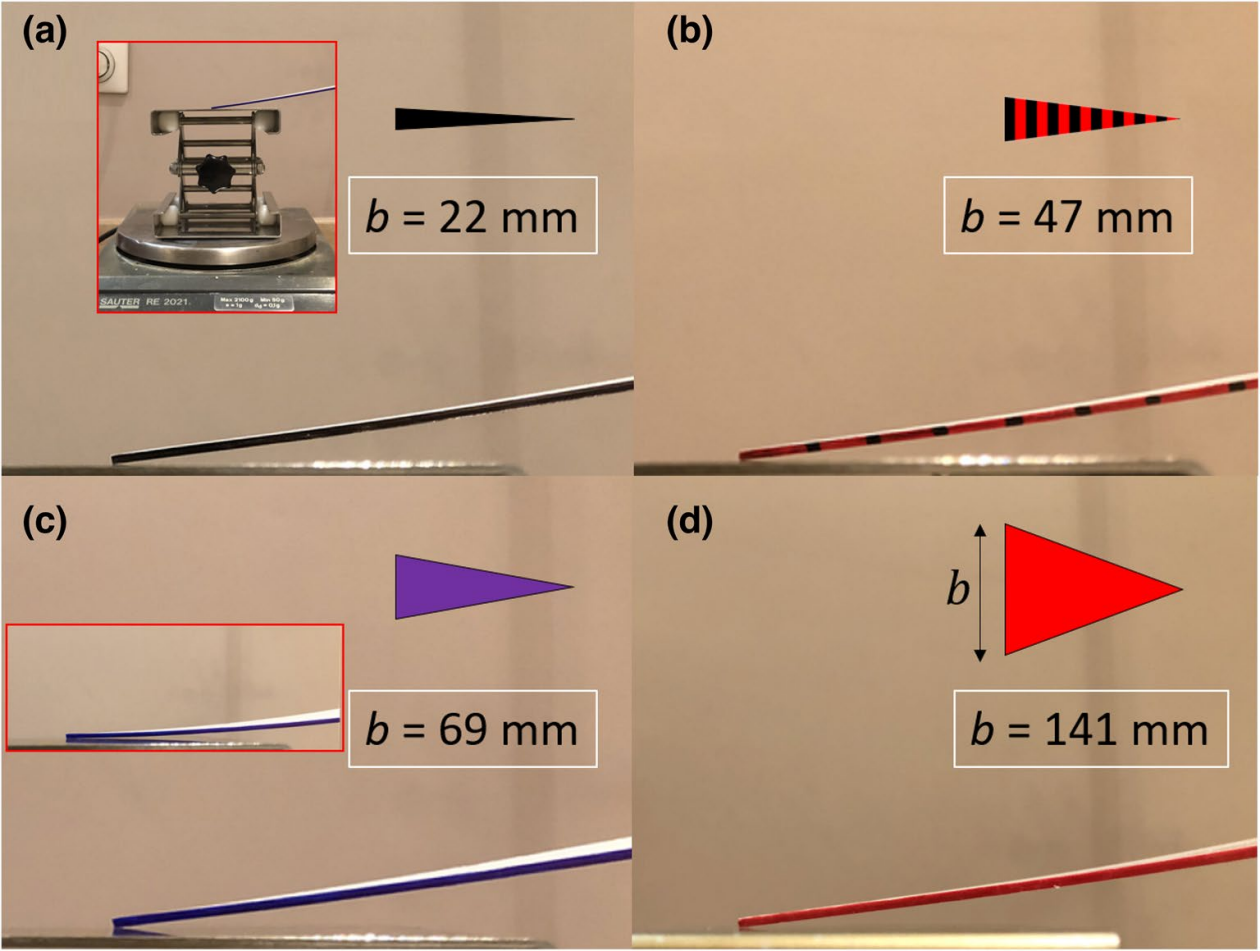
Photographs showing the contact force versus overtravel experiments. The tips of the triangular cantilevers are in contact with the adjustable height laboratory stand (indicated in the inset to (**a**). Photographs are shown for a triangular cantilever base width $b$ of: (**a**) 22 mm, (**b**) 47 mm, (**c**) 69 mm, and (**d**) 141 mm. The inset to (**a**) shows the experimental setup. The inset to (**c**) shows the tip in tangency with the surface.

Figure 6 shows the tip of the triangular cantilevers in contact with the surface of the adjustable height support which itself is resting on the scales to enable a measurement of the force (${F}_{mg}=mg$). The inset to Fig. 6 shows the experimental setup. Four different cantilevers are shown in the figure. The adjustable height support was raised until the tip achieved tangency—this gave the maximum force—see inset to Fig. 6c.

**Figure 7:**
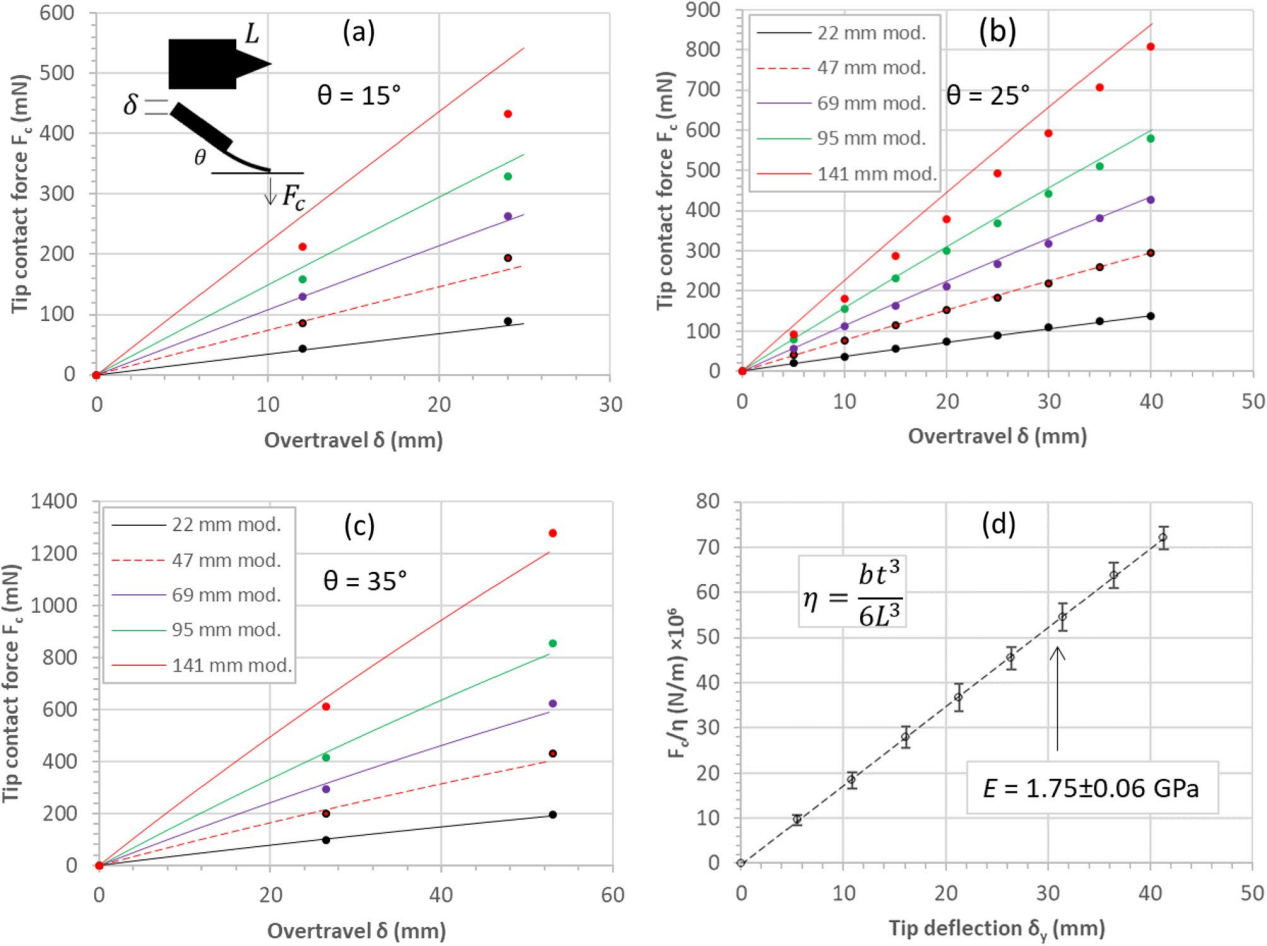
Experimental contact force versus overtravel for triangular cantilevers bending and skating on a flat surface due to an imposed overtravel. (**a**) Tip contact force versus overtravel for a cantilever tilt angle of (**a**) 15°, (**b**) 25°, and (**c**) 35°. (**d**) The factor $F/\eta$ of all data plotted as a function of tip deflection. The traces (solid and dashed) correspond to the mathematical model developed in “Methods”.

Figure 7 shows the results of the contact force versus overtravel experiments. The contact force versus overtravel demonstrated a linear relationship—even up to the tip tangency condition. This was the case for the three tilt angles considered: 15°—Fig. 7a, 25°—Fig. 7b, and 35°—Fig. 7c. The experimental data enabled a plot of the normalised contact force (${F}_{c}/\eta$) to be plotted as a function of tip deflection ${\delta }_{y}$—see Fig. 7d. This enables the elastic modulus $E$ of the polystyrene to be accurately evaluated to be 1.75 ± 0.06 GPa. The traces in Fig. 7 are generated using the mathematical modelling developed in “Methods”. The modelling agrees reasonably well with the experimental data apart from quasi-equilateral cantilever, i.e. $b$ = 141 mm.

### Deflection of triangular cantilevers with concentrated load at tip

**Figure 8:**
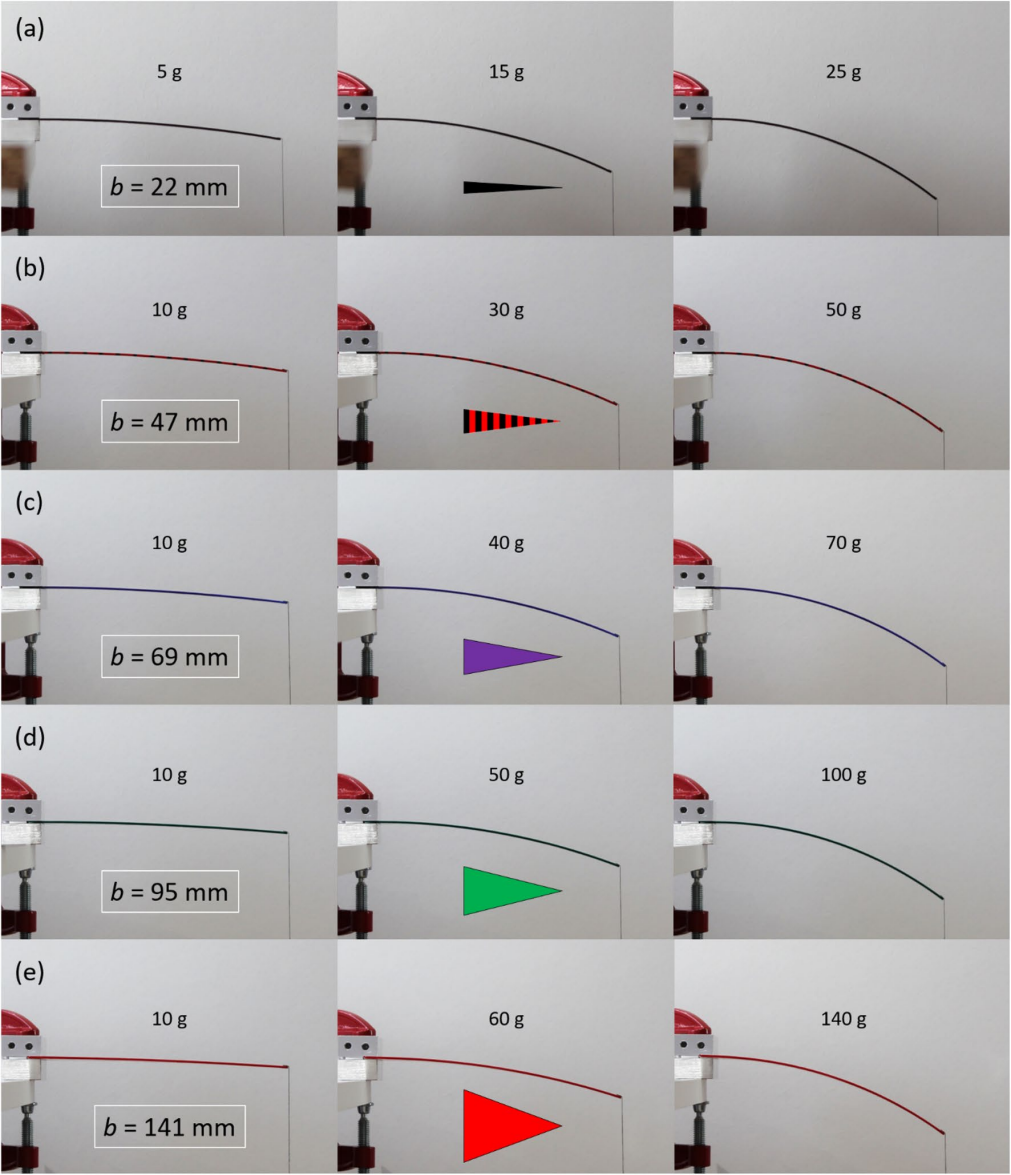
Photographs of the bending of the triangular cantilevers due to a concentrated load at the tip. Results are shown for triangular cantilever base width $b$ of (**a**) 22 mm, (**b**) 47 mm, (**c**) 69 mm, (**d**) 95 mm, and (**e**) 141 mm. In each case the length of the triangular cantilever is 190 mm.

Figure 8 shows photographs of the bending of the triangular cantilever experiments. Photographs are shown for three different weights for five cantilevers having a base width varying from 22 mm—Fig. 8a to 141 mm—Fig. 8e. The largest weight in each case suggests that the bending in high deflection is quasi-circular—indeed, this was demonstrated in the analysis of the images where arcs of circles fitted the bending very accurately. All deflection versus concentrated load experimental results in the form of photographs can be found in the Supplementary Information (see Movies 6–10).

**Figure 9:**
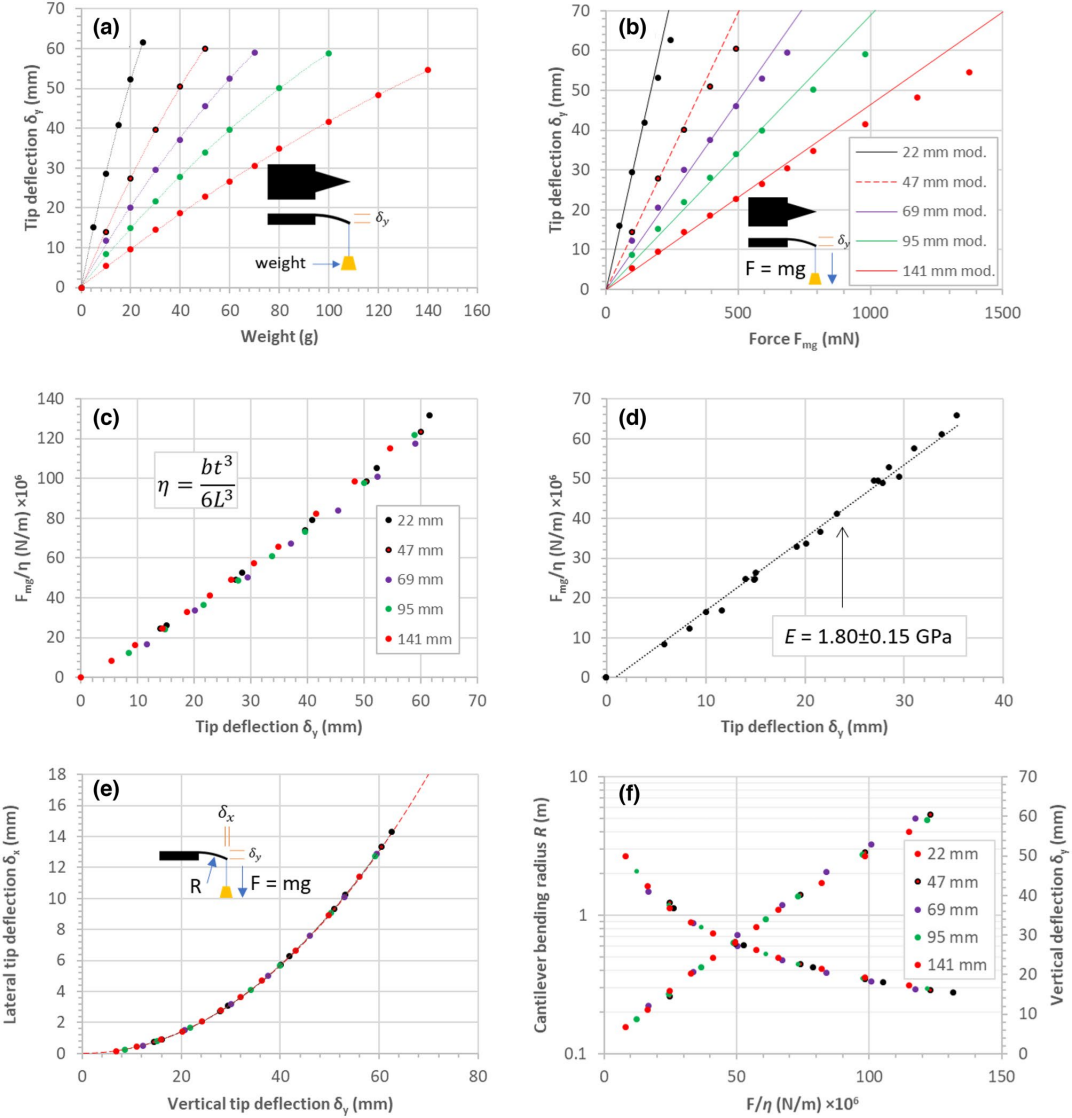
Experimental results of triangular cantilevers bending due to a concentrated load at the tip. (**a**) Tip deflection versus weight. (**b**) Tip deflection versus concentrated load force. (**c**) The factor $F/\eta$ of all data plotted as a function of tip deflection. (**d**) Plot of the linear portion of graph (**c**). (**e**) Plot of the lateral tip deflection versus the vertical tip deflection for all data. (**f**) Plots of the cantilever bending radius and the vertical tip deflection versus the factor $F/\eta$ for all data.

Figure 9 shows the results of the analysis of the data generated from the bending due to a concentrated load at the tip of the cantilever experiments.

In each case, the tip deflection increases with weight—Fig. 9a. The dashed traces in Fig. 9a are parabolic fits. The tip deflection varies linearly with load force up to a certain force—Fig. 9b. The modelling fits (solid lines) the data in the linear portion of the results—Fig. 9b. When the normalised load force (${F}_{mg}/\eta$) is plotted as a function of tip deflection (${\delta }_{y}$)—Fig. 9c, the linear portion of this data enables the elastic modulus to be evaluated to be 1.80 ± 0.15 GPa—Fig. 9d. This agrees well with the value of the elastic modulus obtained from the contact force experiments. Analysis of the photographic data enables the lateral tip deflection (${\delta }_{x}$) to be plotted as a function of the vertical tip deflection (${\delta }_{y}$)—Fig. 9e. The dashed red line in Fig. 9e is based on the model proposed by the author developed in “Methods”. Finally, analysis of the photographic data strongly suggested that the bending of all the triangular cantilevers is circular—even at high deflection—Fig. 9f.

**Figure 10:**
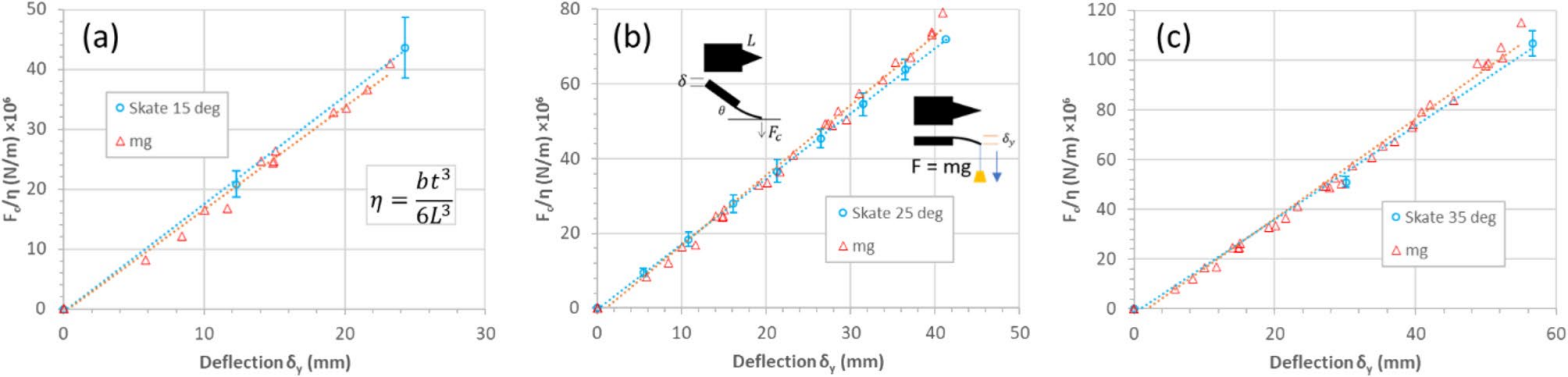
Comparison of cantilever deflection due to concentrated loading (red triangles) and overtravel/skate (blue circles) experiments. The plots correspond to cantilever tilt angles of (**a**) 15°, (**b**) 25°, and (**c**) 35° in the case of skate/overtravel.

Figure 10 shows plots of the comparison of the normalized contact and load forces as a function of tip deflection. This shows that the elastic modulus can be accurately extracted using both experimental methods.

## Modelling

The following section will describe the modelling developed here for the understanding and prediction of skate and contact force in tilted triangular cantilevers in contact with a surface and bending due to an imposed overtravel. First, a relationship between the lateral and vertical tip deflection will be developed by assuming circular bending, as was observed experimentally. Second, this relationship will be used to predict the tip skate of a triangular cantilever due to an imposed overtravel—a comparison will be made with the rectangular cantilever case^11^. Third, the contact force between the tip of the triangular cantilever and the surface will be modelled.

### Beam theory

**Figure 11:**
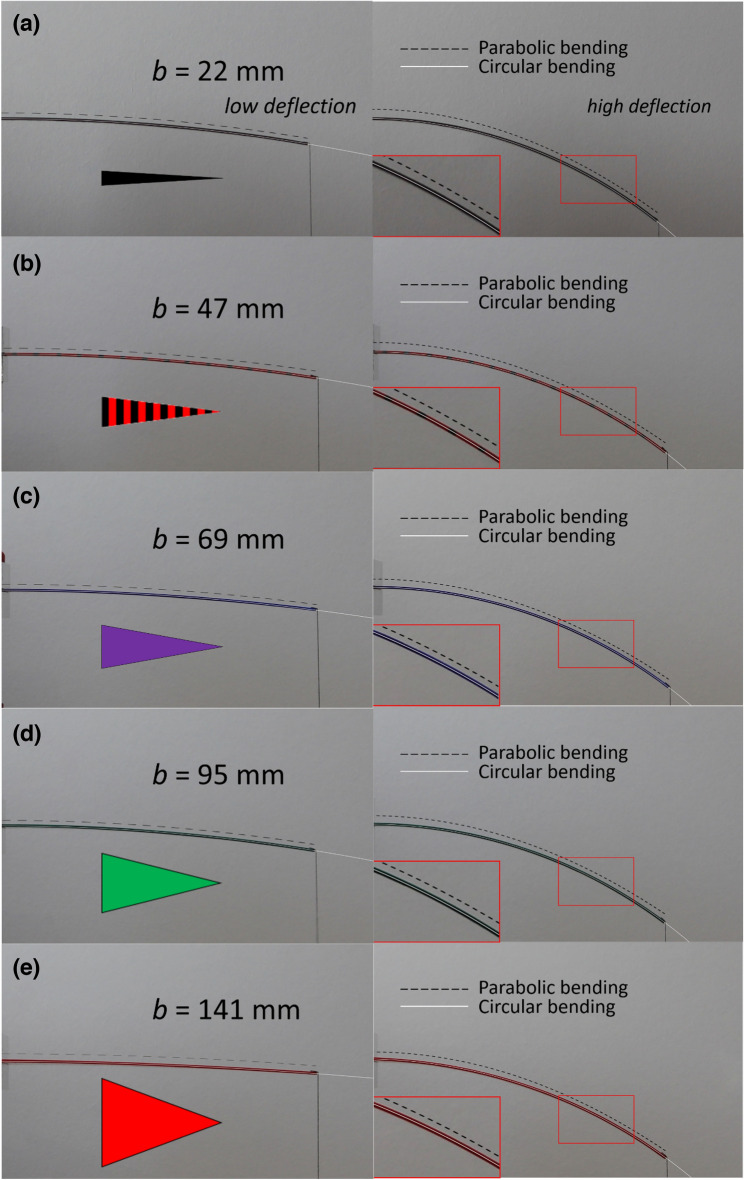
Bending of triangular cantilevers in high deflection. The base widths $b$ are 22 mm, 47 mm, 69 mm, 95 mm, and 141 mm. The length of the cantilevers $L$ is 190 mm the thickness $t$ is 1.5 mm. The dashed black curve shows the solution of the EB equation, the white curves show circular arcs. The insets on right-had photos (high deflection) show a zoom illustrating a circular arc fit (white line) and the parabolic bending model (dashed black lines).

Beam theory, based on the Euler–Bernoulli equation, predicts a parabolic deflection of a triangular cantilever for a concentrated load at the apex of the cantilever (see Supplementary Information). The experimental results indicate a quasi-circular bending of the triangular cantilevers in high deflection—see Fig. 11. This is the deflection regime where flexible MEMS cantilevers could be used to achieve low contact resistance and tip planarity. The dashed black curves on Fig. 11 show the solution of the EB equation—the white curves on Fig. 11 show circular bending. At low deflection the experimental deflection can be modelled by the parabolic solution. However, it is apparent that circular arcs fit the experimental data better at high deflection. Thus, in order to model the overtravel/skate/contact force relationship of a probe based on a triangular cantilever undergoing high deflection, a circular bending can be assumed to provide an approximate solution. This approach was taken previously to approximate a rectangular cantilever by using a parabolic bending^11^ to provide an analytical solution for the deflected cantilever’s arc length. This enables an expression for the lateral deflection of the cantilever tip to be found, which enables an expression for the overtravel versus skate relationship to be obtained. Note that a treatment using Timoshenko beam theory is not necessary here as the Timoshenko condition is not met (see Supplementary Information). However, if greater accuracy was sought, a better approximation to the current problem would be possible using a modified Euler–Bernoulli approach for high deflection of beams, but this approach would necessitate a numerical solution (see Supplementary Information).

### Bending and tip deflection of a triangular cantilever under high deflection

First, a relationship between the vertical deflection ${\delta }_{y}$ and the lateral deflection ${\delta }_{x}$ for a circularly-bending triangular cantilever can be found in the following way. The length $s$ of the sagitta of a circular arc can be approximated by the following expression:

1 $$s=\frac{{c}^{2}}{8R}$$

where $c$ is the chord length and $R$ is the radius of curvature of the arc. This expression is valid when $s\ll R$. The arc length $L$ is approximated by the following formula:

2 $$L=c+\frac{8{s}^{2}}{3c}$$

Therefore:

3 $$L=c\left(1+\frac{{c}^{2}}{24{R}^{2}}\right)$$

We know that $c=\sqrt{{{x}_{0}}^{2}+{{y}_{0}}^{2}}$; where ${x}_{0}$ and ${y}_{0}$ are the coordinates at the end of the circular arc. It is therefore straightforward to fix the length $L$ at unity and vary the radius of curvature $R$ for the circular arc and numerically find the values of the coordinates ${x}_{0}$ and ${y}_{0}$ at the end of the deflected cantilever of length unity using the following equation:

4 $$\sqrt{{{x}_{0}}^{2}+{{y}_{0}}^{2}}+\frac{{\left({{x}_{0}}^{2}+{{y}_{0}}^{2}\right)}^\frac{3}{2}}{24{R}^{2}}-1=0$$

**Figure 12:**
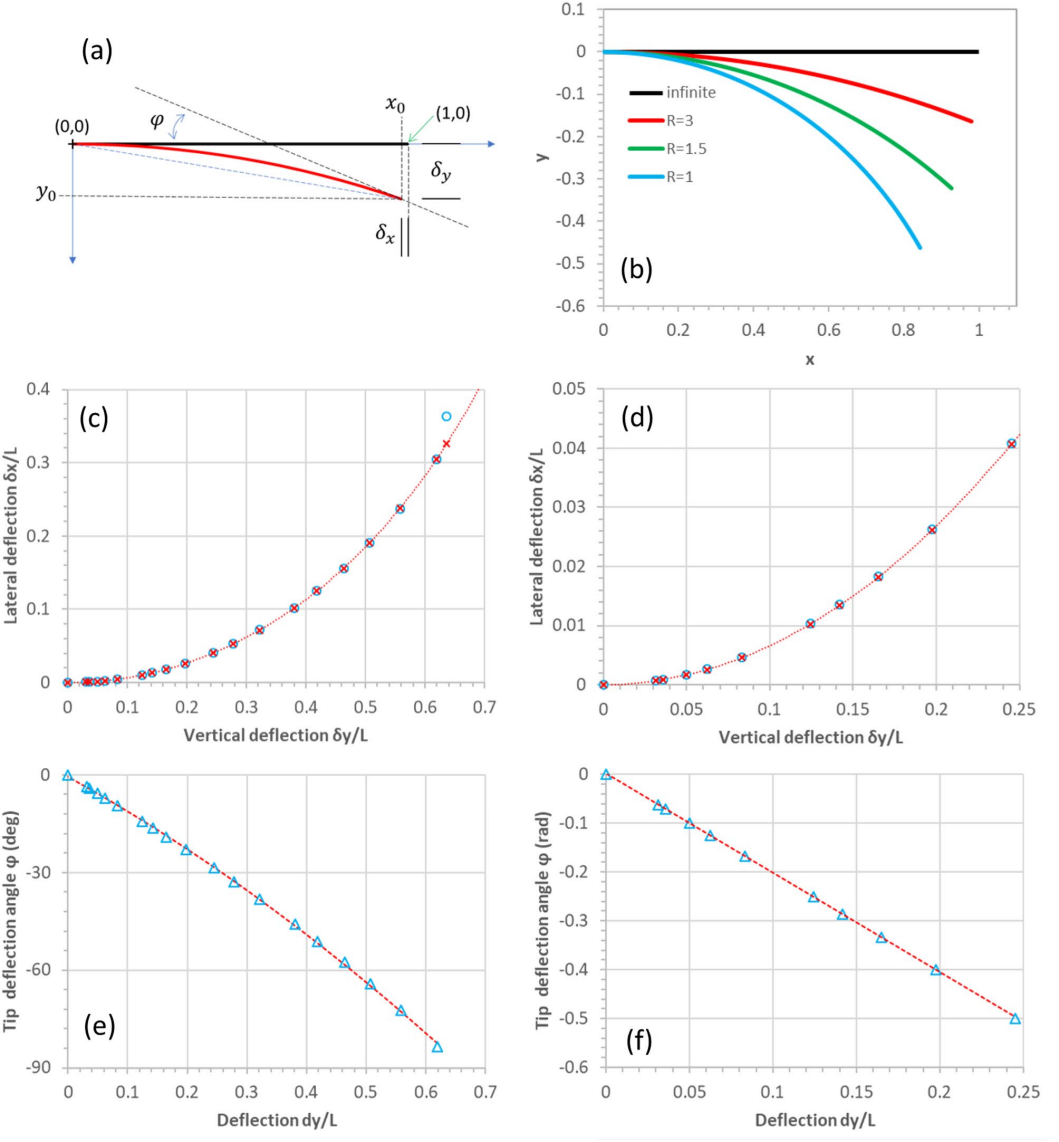
Modelling of a triangular cantilever bending in high deflection. (**a**) Schematic diagram showing the side view of a deflecting triangular cantilever under a concentrated load at the tip. (**b**) The modelled profile of the triangular cantilever of unit length undergoing deflection at various bending radii. (**c**) The modelled lateral deflection of the tip as a function of vertical deflection. (**d**) A zoom of graph (**c**). (**e**) The tip deflection angle versus the tip deflection. (**f**) A zoom of graph (**e**) showing the quasi-linear region.

The deflections at the end of the cantilever are therefore given by ${\delta }_{y}={y}_{0}$ and ${\delta }_{x}=1-{x}_{0}$. The numerically-obtained values of ${\delta }_{y}$ and ${\delta }_{x}$ are plotted in Fig. 12b.

It has been verified that to find the deflection lengths ${\delta }_{y}$ and ${\delta }_{x}$ of a cantilever of length $L$, one must simply multiply ${\delta }_{y}$ and ${\delta }_{x}$ by $L$.

The data points (blue circles) in Fig. 12c,d are generated from the above modelling. The data points (red crosses) and traces (dashed red lines) are generated using modelling developed in the following section.

### Skate versus overtravel in triangular cantilevers

**Figure 13:**
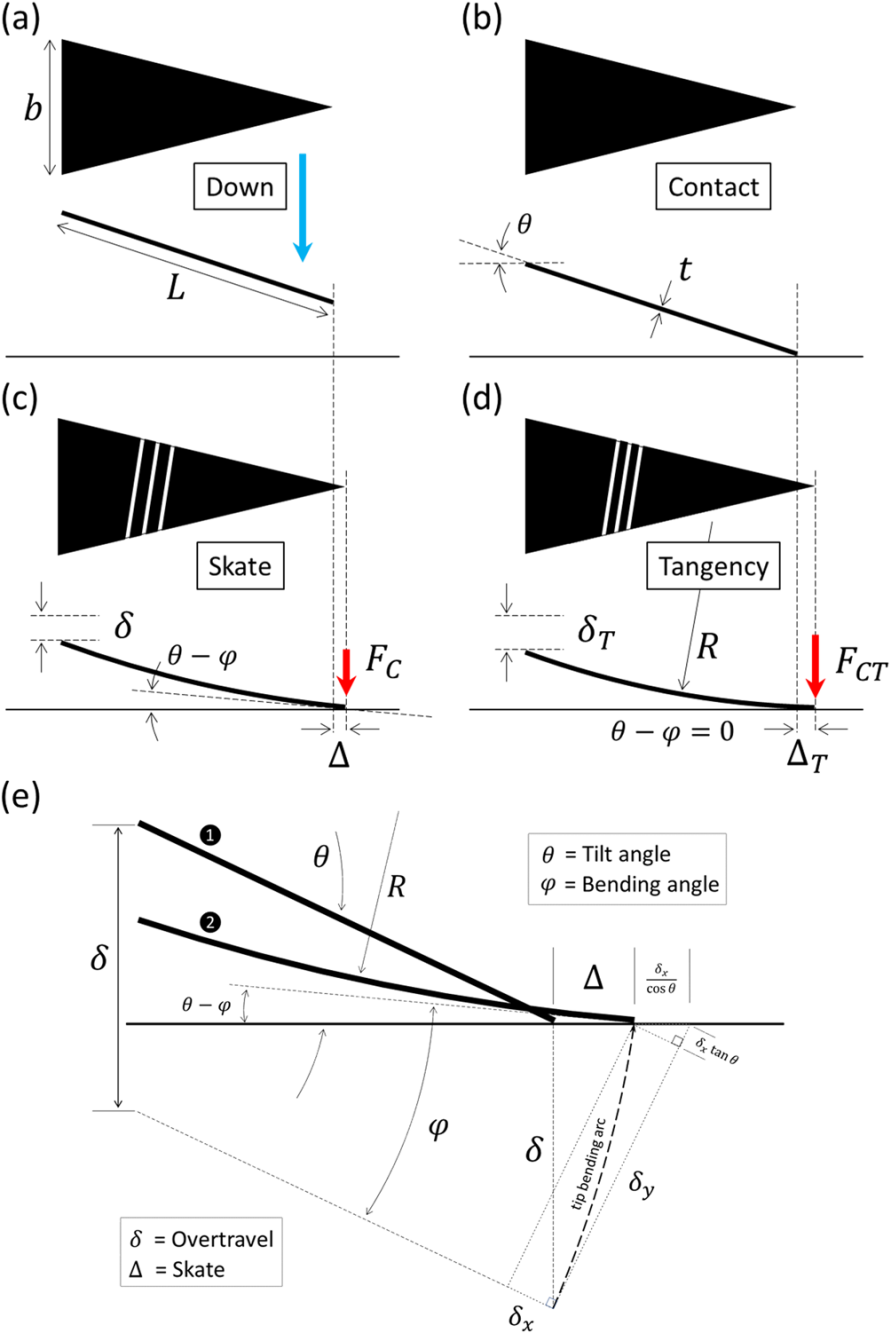
The geometry of the tip of the triangular cantilever skating on a surface due to an imposed overtravel. The model considers a circular bending of the triangular cantilever—this was experimentally verified. The dashed arrow indicates the bending arc of the tip when it skates across the surface due to the imposed overtravel. (**a**) The tilted triangular cantilever descending vertically towards the surface. (**b**) the cantilever tip makes contact with the surface (commonly known as probe touchdown). (**c**) An imposed overtravel causes the cantilever to bend and the tip of the cantilever to skate across the surface—a contact force is generated between the tip and the surface. (**d**) A ‘tangency overtravel’ causes the tip to be tangential to the surface. (**e**) the geometrical situation at the tip during skating due to an imposed overtravel—the bending is circular for a triangular cantilever.

In order to obtain a formula for the horizontal skate $\Delta$ along the surface in terms of the imposed vertical overtravel $\delta$ we can consider the geometry of the deflecting triangular cantilever shown in Fig. 13.

Elementary trigonometry allows one to obtain the following formulae for the overtravel $\delta$ and the skate $\Delta$ in terms of the and the probe angle $\theta$^11^:

5 $$\delta ={\delta }_{y}\mathrm{cos}\,\theta +{\delta }_{x}\mathrm{sin}\,\theta$$

6 $$\Delta = \delta \mathrm{tan}\,\theta -\frac{{\delta }_{x}}{\mathrm{cos}\,\theta }$$

where ${\delta }_{x}$ and ${\delta }_{y}$ are the movement of the end of the cantilever due to bending by imposing an overtravel—see Fig. 13. Based on the results of numerical modelling of the tip deflection under circular bending shown in Fig. 12 the author proposes the following equation for the lateral movement ${\delta }_{x}$ of a circularly-deflecting triangular cantilever in terms of the tip deflection ${\delta }_{y}$:

7 $${\delta }_{x}=fL\left[\sqrt{\frac{{\mathrm{sin}}^{-1}\frac{{\delta }_{y}}{L}}{\frac{{\delta }_{y}}{L}}}-1\right]$$

where $f$ is a constant equal to 7.929 and $L$ is the length of the cantilever. This relationship is very accurate up to ${\delta }_{y}/L=0.63$, i.e. ${\delta }_{x}/L=0.3$; these are the data points (red crosses) and traces (red dashed lines) shown in Fig. 12.

The bending angle $\varphi$ of the end of the triangular cantilever can be approximated by the following relationship:

8 $$\varphi =\alpha {\left(\frac{{\delta }_{y}}{L}\right)}^{2}+\beta \frac{{\delta }_{y}}{L}+\gamma$$

where $\alpha$, $\beta$, and $\gamma$ are constants evaluated numerically to be 0.912, 1.734, and 0.015. This equation is accurate up to ${\delta }_{y}/L=0.63$, i.e. ${\delta }_{x}/L=0.3$; these are the traces (red dashed lines) shown in Fig. 12. Interestingly, if ${\delta }_{y}/L<0.3$ (i.e. ${\delta }_{x}/L=0.05$) then the following relationship is very accurate—see Fig. 12f:

9 $$\varphi =\frac{2{\delta }_{y}}{L}$$

It is well known in atomic force microscopy that the tilt angle $\theta$ of the probe leads to an apparent increase in the spring constant of the cantilever by 10–20%^15^. Modelling suggests that this can be accounted for in a rectangular cantilever by applying a factor ${\mathrm{cos}}^{2}\theta$^9,10,16^. This has recently been verified experimentally by Gates^17^ for probe angles up to 15° and, importantly, small tip deflection. This may not be the case for electrical contacting probes based on microcantilevers where tangency of the tip may be sought to achieve low resistance electrical contacts between the tip and metal pads on the surface. In this case the deflection of the microcantilever will be high and the ${\mathrm{cos}}^{2}\theta$ compensation factor may no longer be valid for the force deflection relationship. In an effort to understand this relationship I have conducted experiments on macroscopic triangular cantilevers. It has been shown previously that the measurement of macroscopic measurements can contribute an understanding to micrometric devices^11,18^. All this work has been done in the context of very low deflection AFM cantilevers. This is in contrast to the work here in the electrical probes where the defection can be relatively high. In this case empirical evidence suggests a cos theta cubed relationship.

The author has explored the validity of these relationships experimentally—see experimental “Modelling”. For a long, thin cantilever, i.e., $L\gg w$, Eqs. (7) and (8) describe a fixed-free cantilever’s lateral deflection and bending angle accurately for deflections up to $L/3$. For a fixed-free cantilever bending under a concentrated load located at the free end, the following conditions indicate the linear and nonlinear behaviours: $\varphi /\mathrm{sin}\varphi \cong 1$ (linear) and $\varphi /\mathrm{sin}\varphi \gg 1$ (nonlinear)—where $\varphi$ is the bending angle of the end of the cantilever. Substituting ${\delta }_{y}=L/3$ into Eq. (9) gives $\varphi /\mathrm{sin}\varphi =1.04$.

**Figure 14:**
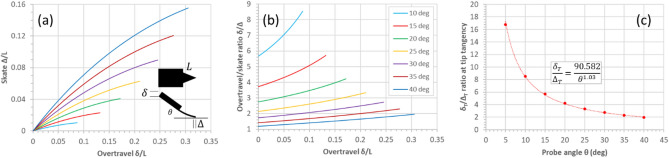
Universal skate/overtravel curves for tilted triangular cantilevers bending in contact with a surface due to an imposed overtravel. (**a**) The skate versus the overtravel as a function of cantilever tilt angle. (**b**) The overtravel/skate ratio as a function of cantilever tilt angle. (**c**) The tangency overtravel/stake ratio versus the cantilever tilt angle.

Figure 14 shows useful graphs which can be used to evaluate the skate and overtravel-to-skate ratio of a triangular cantilever having a length $L$—the relationships are independent of the base width $b$ and the thickness $t$. For example, if the triangular cantilever has a length of 100 µm and the overtravel is 20 µm, then according to Fig. 14a the skate (for a tilt angle of 30°) is 8 µm. Tip tangency for this cantilever is achieved at an overtravel of ~ 24 µm, resulting in a skate of ~ 9 µm.

**Figure 15:**
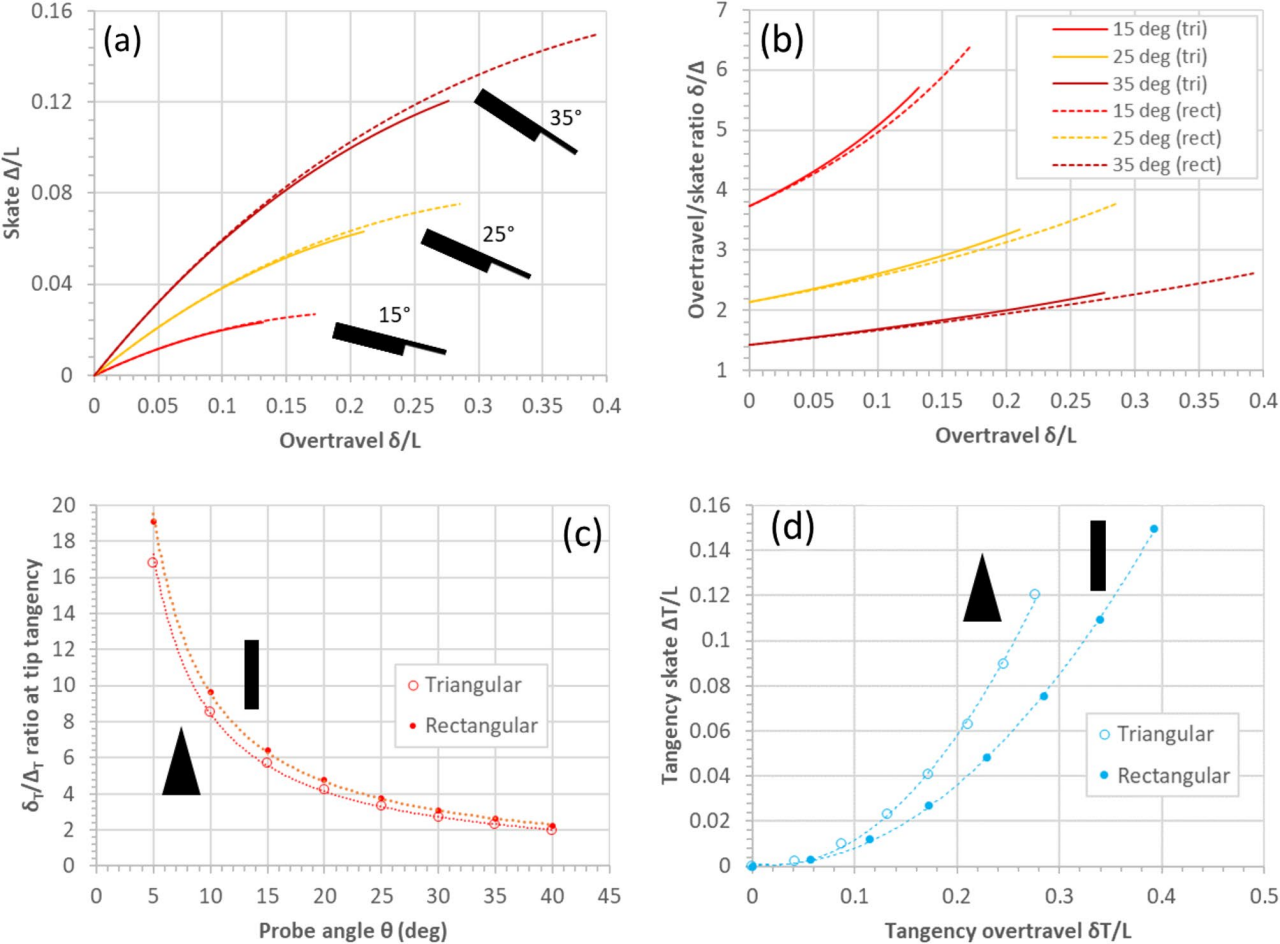
Comparison of skate and overtravel between a triangular cantilever and a rectangular cantilever. (**a**) The skate versus overtravel for triangular (solid curves) and rectangular (dashed curves) cantilevers. (**a**) The overtravel/skate ratio versus overtravel for triangular (solid curves) and rectangular (dashed curves) cantilevers. The tangency overtravel/skate ratio versus the cantilever tilt angle for triangular (open circles) and rectangular (filled circles) cantilevers. The tangency skate versus the tangency overtravel for triangular (open circles) and rectangular (filled circles) cantilevers. All graphs (solid or dashed) correspond to the modelling.

Figure 15 illustrates the comparison between the skate/overtravel relationship for a triangular cantilever (solid curves) and a rectangular cantilever (dashed curves). The modelling indicates clearly that a larger overtravel must be imposed on a rectangular cantilever than a same-length triangular cantilever to achieve tip tangency with the surface—Fig. 15a. In addition, the tip overtravel/skate relationships are slightly larger for a given overtravel—Fig. 15b. For a given tilt (probe) angle, the tangency overtravel-to-skate ratio is smaller for a triangular cantilever in comparison to a rectangular one—Fig. 15c. Figure 15d shows the tangency skate versus overtravel relationships for a triangular and rectangular cantilever.

### Contact force between the tip and the surface

For a triangular cantilever, the relationship between the concentrated force at the tip $F$ and the tip deflection ${\delta }_{y}$ is given by:

10 $$F=\frac{{\delta }_{y}E{t}^{3}b}{6{L}^{3}}$$

where $E$ is the elastic modulus of the material, $L$, $b$, and $t$ are the length, base width, and thickness of the triangular cantilever (see Supplementary Information). Equation (10) is used to model the experimental data presented in Figs. 7 and 9. This models the measured data relatively well for a large range of tilt angles and cantilever base widths. In practice, triangular shaped cantilever-based MEMS probes are likely to have a trapezoidal shape with a finite tip length to accommodate electrical contacts. The deflection and contact force relationship of such probes is given in the Supplementary Information The modelling could therefore be extended to accommodate this and the presence of contact pads having a finite thickness.

**Figure 16:**
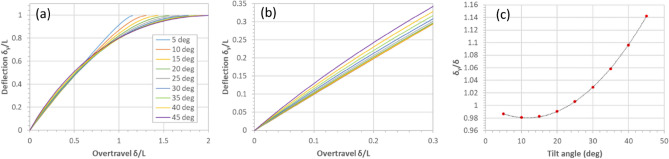
The relationship between overtravel and vertical deflection of the tip of a tilted triangular cantilever. (**a**) tip deflection as a function of imposed overtravel for various tilt angles. (**b**) the quasi-linear portions of the curves. (**c**) the tip deflection/overtravel ratio as a function of the cantilever tilt angle.

Finally, Fig. 16 shows the relationship between the imposed overtravel and the vertical deflection of the tip of a triangular cantilever tilted at a certain angle relative to the surface. Figure 16a shows that these relationships are not linear. However, if we restrict ourselves to an overtravel ($\delta /L$) of 0.3, then the plots are quasi-linear—see Fig. 16b. This enables the plot shown in Fig. 16c which can be used to compute the value of ${\delta }_{y}$ in Eq. (10) at a given tilt angle for a given overtravel $\delta$—as it is the latter which is of practical interest. The plot shown in Fig. 16c can be very accurately fitted with a quadratic equation to be ${\delta }_{y}/\delta =0.477{\theta }^{2}-0.195{\theta }+1$, if $\theta$ is in radians. Note that for a triangular cantilever tilted at ~ 23.4° then the tip deflection is equal to the overtravel, provided that $\delta /L\le 0.3$.

### Skate compensation in tapered probes

**Figure 17:**
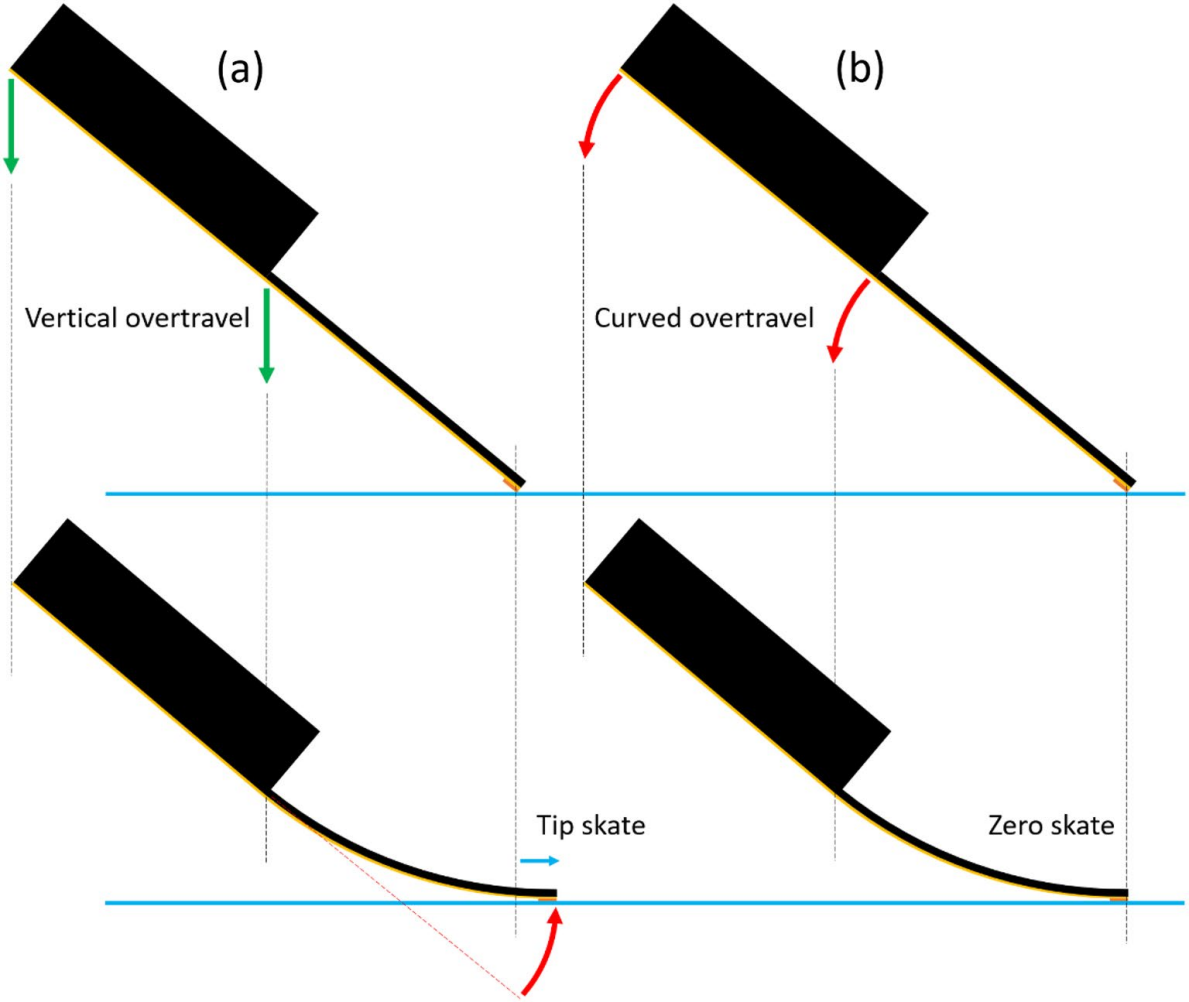
Zero skate of tapered probes based on a triangular cantilever achieved using skate compensation. (**a**) Vertical overtravel (green arrows) of the rigid support chip results in tip skate (blue arrow) when the tip of the cantilever follows an arc (red arrow). (**b**) Curved overtravel (red arrows) of the rigid support chip results in zero skate.

The findings here indicated that a practical skate-compensation is feasible for triangular-shaped, tapered probes to achieve zero skate. This idea was introduced for probes based on rectangular microcantilevers^11^. To do this, the overtravel should not be vertical but rather follow the arc of the tip deflection, i.e. the ${\delta }_{y}$ versus ${\delta }_{x}$ relationship given by Eq. (7). Figure 17 shows schematically the idea of attaining zero-skate for a tapered probe—this could be accomplished using a specially-designed mechanical part or stepper motors.

## Conclusions

An understanding of the interplay between the overtravel, the skate, and the tip-to-surface contact force of a tilted triangular cantilever in the high-deflection regime may be of some importance for emerging MEMS microcantilever-based chip-edge probe technologies fabricated using microtechnology. To achieve this, macroscopic experiments can be of use to predict the behaviour of micrometre-size probes—where experimental work on the latter may be more challenging. Indeed, a robust macroscopic experimental setup is shown to lead to excellent measurement repeatability. Macroscopic cantilevers have been fabricated using polystyrene sheets. The elastic modulus of dense polystyrene means that centimetre dimensions can be used for the cantilevers’ lateral dimensions. The dimensions of the macroscopic cantilevers can be chosen so as to give typical cantilever stiffnesses in the range of commercial silicon microcantilevers—this facilitates the comparison of the experimental results with the predictions of the model for future microfabricated microscopic objects. Consequently, a macroscopic approach enables scalable models to be easily tested, and with relative accuracy. Modelling enables universal scalable relationships between skate, overtravel, and contact force to be developed; such models should be of use to the engineer. These relationships should be valid for micrometre-sized cantilevers as the approach is, at least in principle, scalable. An accurate relationship between the lateral and vertical deflection of the tip of a triangular cantilever under high deflection is proposed by the author—this is based on both experimental evidence and modelling. The model successfully predicts the experimentally-obtained skate versus overtravel data. Interestingly, compared to a rectangular cantilever, less overtravel is required to achieve the tangency overtravel/skate condition for a triangular cantilever—this is due to the circular bending of a triangular cantilever rather than the cubic polynomial bending of a rectangular cantilever. This has implications for the overtravel/tip-to-surface contact force relationship. It is found experimentally that the skate/overtravel relationship is independent of triangular base width—this has been experimentally validated over a $b/L$ range of 0.12 to 0.74, i.e. from strongly-isosceles to quasi-equilateral. This means that the skate/overtravel relationship of a triangular cantilever is independent of the cantilever stiffness. There is a relationship between the contact force and the concentrated load force at the tip which causes bending under high deflection. The measured tip/surface contact force increases linearly as a function of triangular cantilever base width. The contact force depends on the triangular base width. The bending of a triangular cantilever means that there is therefore no stress concentration at the base unlike in rectangular cantilevers; this may be an advantage in terms of the robustness of future microcantilevers. The tangency condition in triangular cantilevers is reached earlier (i.e. for less overtravel) than in rectangular cantilevers having the same length due to circular bending—this may be an advantage in terms of electrical contacting. The findings have also allowed the suggestion of a practical solution to achieve zero skate in tapered MEMS probes. Finally, one can conclude that the findings here concerning the interplay between the overtravel, the skate, and the tip-to-surface contact force of large-deflection tilted triangular cantilevers may be of some importance for emerging MEMS microcantilever-based probe technologies fabricated using microtechnology which function in the high-deflection regime.

## Methods

### Skate versus overtravel for tilted triangular cantilevers

The skate versus overtravel relationship for the five different triangular cantilevers orientated at different tilt angles (15°, 25°, and 35°) was experimentally obtained using an in-house fabricated mechanical part which enabled the cantilever to be inclined relative to a vertically-oriented surface. To minimize friction, a polystyrene sheet was employed as the tip contacting surface and inclined vertically so that the cantilever overtravel could be applied by accurately sliding the mechanical part horizontally on a flat surface. The skate was measured using a precision steel ruler and ‘macro’ photography (Canon 1300D). Again, photography was also used (same conditions as described above) to obtain side-view images of the cantilever bending for later analysis of the cantilever bending. As above, these photographs enabled an accurate determination of the curvature of the cantilevers during overtravel and skating. The overtravel was measured using a precision steel ruler and ‘macro’ photography—the steel ruler also acted as a physical guide for the sliding mechanical part holding the tilted cantilever.

### Contact force versus overtravel for tilted triangular cantilevers

The tip/surface contact force was obtained by the bending triangular cantilever pushing down on precision scales—this mimicking a tilted microcantilever probe. To do this, the triangular cantilevers were mounted onto a laboratory stand and a clamp. The clamp enabled the cantilever to be oriented at an angle of 15°, 25°, and 35° relative to the horizontal. The overtravel was applied using a variable-height stand present on the scales whose weight was subtracted (by zeroing the scales). The stable setup provided excellent repeatability in the measurement of the contact force as a function of overtravel. A precision scales (Sauter GMbH, Germany) accurate to ± 0.1 g was used to evaluate the contact force of the triangular cantilever under imposed overtravel. The contact force (in newtons) is calculated by multiplying the ‘weight’ measured by the scales by the acceleration due to gravity $g$ 9.81 ms^−2^. The triangular cantilevers were mounted above the precision scales using a laboratory stand, clamps, and a bench clamp. In order to impose an overtravel, a small adjustable height (5–15 mm) laboratory support was used-the accuracy of the height was ± 0.5 mm. Highly reproducible measurements were obtained using the setup.

### High-deflection bending of a triangular cantilever due to concentrated load at tip

The relatively high stiffness of the cantilevers (ranging from 3.3 N m^−1^ for a base width of 22 mm to 20.9 N m^−1^ for a base width of 141 mm) along with their low weight (due to the low density of polystyrene, measured to be approximately 1042 kg m^−3^) means that bending due to gravity at zero tip loading is negligible. The triangular cantilevers were thus clamped horizontally, and precision weights were suspended from their tips using a carefully-glued cotton attached to the cantilever tips (also negligible weight)—this can be seen in Fig. 1. The vertical deflection of the cantilevers was initially measured using a vertically-orientated precision steel ruler. Photography (a DSLR Canon EOS 1300D with an EFS 28–55 mm zoom lens in manual fixed focus mode mounted on a tripod and position at a distance of approximately 2 m to achieve perspective control and an aperture (*f*5.6) for enough depth of field enabled side-view images of the cantilever bending to be obtained for accurate image analysis. The curvature of the bending cantilevers, along with the vertical and lateral deflection of the cantilever tips, were very accurately determined from these photographs. Using maximum zoom, an aperture size of *f*5.6 was found to achieve enough depth of focus for a sharp cantilever edge. The ISO was set to 200 given the room lighting conditions—resulting in photographs with minimal graininess.

## Supplementary Information

Supplementary Legends.

Supplementary Movie 1.

Supplementary Movie 2.

Supplementary Movie 3.

Supplementary Movie 4.

Supplementary Movie 5.

Supplementary Movie 6.

Supplementary Movie 7.

Supplementary Movie 8.

Supplementary Movie 9.

Supplementary Movie 10.

Supplementary Information 1.

## Data Availability

All data generated or analysed during this study are included in this published article.
