# Supplementary material for: Skate, overtravel, and contact force of tilted triangular cantilevers for microcantilever-based MEMS probe technologies
Source: Sci Rep. 2022 Nov 12;12:19386. doi: 10.1038/s41598-022-23973-5 (PMC9653427; doi:10.1038/s41598-022-23973-5)
Supplement: Supplementary file 1 — Supplementary Legends. [file 41598_2022_23973_MOESM1_ESM.docx]

**Legends for Supplementary Movies**

Movie1. Skate versus overtravel for a triangular cantilever having a length of 190 mm, a thickness of 1.5 mm, and a base width of 22 mm. The tilt angle is 25 degrees.

Movie2. Skate versus overtravel for a triangular cantilever having a length of 190 mm, a thickness of 1.5 mm, and a base width of 47 mm. The tilt angle is 25 degrees.

Movie3. Skate versus overtravel for a triangular cantilever having a length of 190 mm, a thickness of 1.5 mm, and a base width of 69 mm. The tilt angle is 25 degrees.

Movie4. Skate versus overtravel for a triangular cantilever having a length of 190 mm, a thickness of 1.5 mm, and a base width of 95 mm. The tilt angle is 25 degrees.

Movie5. Skate versus overtravel for a triangular cantilever having a length of 190 mm, a thickness of 1.5 mm, and a base width of 141 mm. The tilt angle is 25 degrees.

Movie6. Deflection versus weight for a triangular cantilever having a length of 190 mm, a thickness of 1.5 mm, and a base width of 22 mm.

Movie7. Deflection versus weight for a triangular cantilever having a length of 190 mm, a thickness of 1.5 mm, and a base width of 47 mm.

Movie8. Deflection versus weight for a triangular cantilever having a length of 190 mm, a thickness of 1.5 mm, and a base width of 69 mm.

Movie9. Deflection versus weight for a triangular cantilever having a length of 190 mm, a thickness of 1.5 mm, and a base width of 95 mm.

Movie10. Deflection versus weight for a triangular cantilever having a length of 190 mm, a thickness of 1.5 mm, and a base width of 141 mm.
