# Supplementary material for: Skate, overtravel, and contact force of tilted triangular cantilevers for microcantilever-based MEMS probe technologies
Source: Sci Rep. 2022 Nov 12;12:19386. doi: 10.1038/s41598-022-23973-5 (PMC9653427; doi:10.1038/s41598-022-23973-5)
Supplement: Supplementary file 12 — Supplementary Information 1. [file 41598_2022_23973_MOESM12_ESM.docx]

*Supplementary Information for:*

**Skate, overtravel, and contact force of tilted triangular cantilevers for microcantilever-based MEMS probe technologies**

Steve Arscott

University of Lille, CNRS, Centrale Lille, University Polytechnique Hauts-de-France, UMR 8520-IEMN, F-59000 Lille, France. email: [steve.arscott@univ-lille.fr](mailto:steve.arscott@univ-lille.fr)

**The Euler-Bernoulli equation**

The Euler-Bernoulli static beam equation is:

$$\frac{d}{dx^{2}}\left( EI\frac{d^{2}y}{dx^{2}} \right)=q$$

Where $E$ is the elastic modulus of the beam, $I$ is the second moment of area of the beam, $y$ is the deflection along the beam, and $q$ is the loading.

**Rectangular cantilever**

For a beam having a constant rectangular cross section (width $w$ and thickness $t$) and a concentrated load $q\left( x \right)=F\delta\left( x-L \right)$ at the tip we have the following expressions for the second moment of area $I$, the bending moment $M$, the deflection $y$, the tangent of the bending angle $\tan\theta$, and the surface stress $\sigma$:

$$I=\frac{wt^{3}}{12}$$

$$M\left( x \right)=F(L-x)$$

$$y(x)=\frac{Fx^{2}}{6EI}\left( 3L-x \right)$$

$$\tan\theta(x)=\frac{Fx}{2EI}(2L-x)$$

$$\sigma\left( x \right)=\frac{6F(L-x)}{wt^{2}}$$

The tip deflection $\delta_{y}$ is thus:

$$\delta_{y}=\frac{FL^{3}}{3EI}$$

The tip bending angle is:

$$\tan\theta=\frac{FL^{2}}{2EI}$$

The maximum stress at the base of the cantilever is:

$$\sigma_{max}=\frac{6FL}{wt^{2}}$$

**Triangular cantilever**

Let us now solve the Euler-Bernoulli equation for a triangular beam having a base width $b$ and thickness $t$. The second moment of area $I$in the Euler-Bernoulli equation is not constant along the length of the triangular:

$$I\left( x \right)=\frac{w(x)t^{3}}{12}$$

Where:

$$w\left( x \right)=b\left( 1-\frac{x}{L} \right)$$

The tangent of the bending angle (${dy}/{dx}$) of the triangular cantilever is given by:

$$\frac{dy}{dx}=\frac{1}{E}\int\frac{M(x)}{I(x)}dx$$

$$\frac{dy}{dx}=\frac{12F}{Et^{3}b}\int\frac{L-x}{1-\frac{x}{L}}dx$$

$$\frac{dy}{dx}=\frac{12F}{Et^{3}b}\left( Lx+C_{1} \right)$$

The deflection $y(x)$ is given by:

$$y\left( x \right)=\int\frac{dy}{dx}dx$$

$$y\left( x \right)=\frac{12F}{Et^{3}b}\int\left( Lx+C_{1} \right)dx$$

$$y\left( x \right)=\frac{12F}{Et^{3}b}\left( \frac{L}{2}x^{2}+C_{1}x+C_{2} \right)$$

By applying the boundary conditions ${dy}/{dx}=0$ at $x=0$, and $y\left( x \right)=0$ at $x=0$ we have:

$$\frac{dy}{dx}=\frac{12FL}{Et^{3}b}x$$

$$y\left( x \right)=\frac{6FL}{Et^{3}b}x^{2}$$

The tangent of the bending angle $\theta$ and the deflection at the tip $\delta_{y}$ of a triangular cantilever having a rectangular cross section and a concentrated load$F$ at the tip are given by:

$$\tan\theta=\frac{12FL^{2}}{Et^{3}b}$$

$$\delta_{y}=\frac{6FL^{3}}{Et^{3}b}$$

The uniforms stress on the surface of a triangular cantilever is given by:

$$\sigma=\frac{6FL}{bt^{2}}$$

**A trapezoidal cantilever**

The triangular case can be extended to a trapezoidal case (base width $b$, tip width $a$, and thickness $t$) to give:

$$y\left( x \right)=\frac{2Fx^{2}}{Et^{3}}\left[ \left( \frac{{3b}^{2}+15a^{2}+18ab}{b^{3}+a^{3}+5ab^{2}+5a^{2}b} \right)L-\left( \frac{6a}{4ab+b^{2}+a^{2}} \right)x \right]$$

Where $b$ and $a$ are the base width and tip width of the tapered trapezoidal cantilever

If $a = 0$, we have the triangular case above, if $a=b$, then we have the rectangular case above. This formula could be of use in the case of practical cantilevers as are illustrated in Figure 1 of the article.

**The Timoshenko condition**

$$\frac{3EI}{\kappa L^{2}AG}\ll1$$

Where $E$ is the elastic modulus, $I$is the second moment of area, $\kappa$ is the Timoshenko sheer coefficient, $L$ is the length of the beam, $A$ is the cross-sectional area, and $G$ is the sheer modulus. For a polystyrene beam having the practical dimensions used in the article this condition is met meaning the Euler-Bernoulli approximation is justified.

**The Euler-Bernoulli equation in high deflection**

$$EI\frac{d^{4}w}{dx^{2}}-\frac{3}{2}EA\left( \frac{dw}{dx} \right)^{2}\frac{d^{2}w}{dx^{2}}=q(x)$$

**Bending approximations to enable tip bending formulae**

In a previous article^1^, in order to obtain a formula for the lateral bending of the tip of a rectangular cantilever a parabolic bending approximation was assumed, whereas the actual bending is a cubic polynomial (see above). The reason for this was that there is an analytical solution for the arc length of a parabola. The difference in the current paper concerning a triangular cantilever is that a circular bending agrees well with the experimental data in high deflection, whereas the Euler-Bernoulli theory predicts a parabolic bending. There is also an analytical approximation for the arc length of a circular arc. The following Supplementary Figure compares these approximations for both rectangular and triangular cantilevers. The fits are based on Equation 7 in the paper.


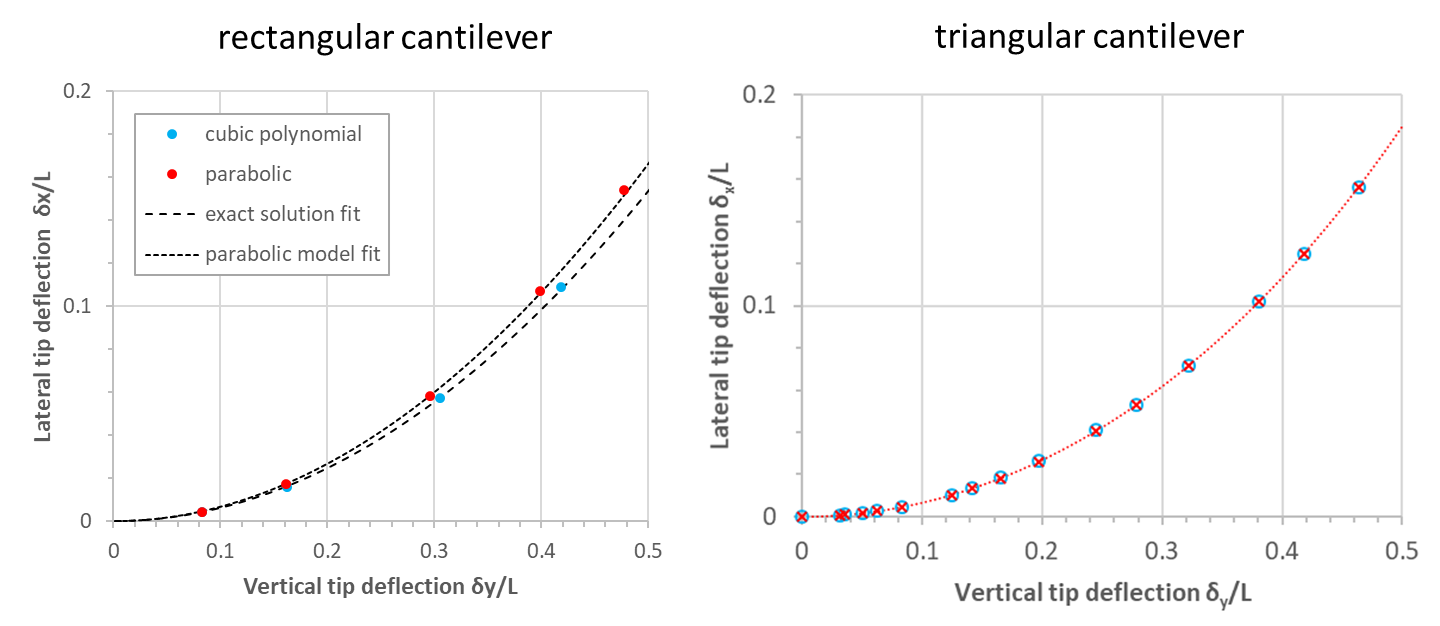


**Supplementary Figure.** Comparison of fits using Equation 7 in the paper. The value of $f$ is 8, 7.4, and 7.9 for a parabolic approximation of a rectangular cantilever, the exact cubic polynomial solution of a rectangular cantilever, and a circular bending of a triangular cantilever.

**Supplementary References**

1. Arscott, S. On overtravel and skate in cantilever-based probes for on-wafer measurements. *J. Micromechanics Microengineering* **32**, 057001 (2022).
